# “Place-finding” as the key to reintegration after release from prison in older age: A transdisciplinary, holistic and strengths-based reintegration framework using grounded theory

**DOI:** 10.1371/journal.pone.0325497

**Published:** 2025-06-17

**Authors:** Ye In Jane Hwang

**Affiliations:** 1 Justice Health Research Program, School of Population Health, Medicine & Health, University of New South Wales, Sydney, New South Wales, Australia; University of Central Lancashire, UNITED KINGDOM OF GREAT BRITAIN AND NORTHERN IRELAND

## Abstract

Prisons globally face an ‘ageing epidemic,’ releasing unprecedented numbers of older adults into the community. Research highlights substantial challenges in reintegrating this underserved and marginalised group. Urgent work is needed to understand their needs and develop effective social, criminological, and public health solutions. This study aimed to create a transdisciplinary, strengths-based conceptual framework to understand and begin addressing the reintegration needs of older individuals leaving prison. Applying grounded theory to qualitative data from Australia – including interviews and workshops with stakeholders and individuals with lived experience – resulted in the development of the ‘Place-finding’ conceptual framework. Successful reintegration can be understood in terms of a journey through stages of “institutionalisation,” “crisis,” “survival & adjustment,” and “grounding”. The framework posits that incarceration produces a ‘loss of place’ in individuals, primarily through institutionalisation and disconnection from society. It argues for the consideration of the right supports at the right time for the unique needs of individuals who will vary in their levels of disconnection. It finds key concepts such as facilitating timely access to services, reconnection with society, building key literacies and healing and restoration from past experiences to be vital in this journey. This preliminary framework offers novel theoretical insights and practical implications for understanding and improving reintegration success in older adults, and is potentially applicable to individuals of various ages, incarceration length and location.

## Introduction

There is a growing number of people being released from prison in middle and older age, for several reasons. First, prison populations worldwide are getting older [1,2]. Several factors contribute to this trend, including general population ageing, increasing sentence length and mass incarceration, and the rise of historical convictions [see 3]. Another influential factor is recidivism rates, which remain high across the world [4]. Resultantly, increasing numbers of people will leave prison in later life than before, often after repeated or lengthy periods of incarceration.

There is consensus that when referring to incarcerated populations, any person who is above 50 years of age should be considered ‘older’ [5]. This is further lowered to 45 for Aboriginal or Torres Strait Islander (Indigenous) peoples [6]. Such a cut-off has been determined due to growing evidence of the intense support needs and often earlier onset of age-related health decline in this population [7–9]. People in contact with the justice system often suffer physical and mental ill-health and socioeconomic disadvantage [10–12], and we can expect that these issues are further compounded with ageing and repeated imprisonment. Ageing in prison populations has been identified by various researchers and stakeholders worldwide as an important emerging issue in the past decade, with significant economic, public health, justice and human rights implications [1,2,13–15].

The present study was motivated by the knowledge and practice gaps surrounding reintegration of people leaving prison in ‘older’ age. In prison, older people have both heightened and unique health and social needs and experiences compared to younger inmates [16–21]. Accordingly, we can expect that post-release needs and experiences will also be unique. Indeed, growing evidence finds more complex health and social needs and unique barriers against receiving support during the post-release period [22–24]. Many practice and policy suggestions have been made, including individual interventions such as health literacy education, as well as broader calls for systemic change in correctional policies, especially in release planning and continuity of care [25–28]. However, key gaps remain.

This study aimed to develop our understanding of the reintegration process after leaving prison in older age, using a Grounded Theory study based in Australia. Australia, like many other countries, is experiencing rapid ageing of its incarcerated population [29,30]. People over the age of 45+ in Australian prisons, who currently represent 1 in every 5 adults, are growing at a much faster rate than younger people [29]. The vast majority of this population are housed in mainstream correctional centres, with only a very limited number of beds dedicated to care for the aged. The vast majority of those incarcerated in Australia will eventually be released [31]. The small but consistent evidence from Australia suggests a range of post-release reintegration challenges for older people that are similar in nature to other Western countries, such as a lack of accessible housing options, a lack of appropriate transition programs and social isolation [32,33].

To our knowledge, there is no clear model or framework for understanding release and reintegration in this age group in literature. Such models are important for bringing together evidence in a structural way and delineating underlying processes for further testing. Conceptual frameworks can also identify the best intervention points and provide direction and priority when implementing solutions [34].

Drawing from available evidence, we selected three distinctive attributes to underpin our construction of a conceptual framework to understand reintegration for people leaving prison at an older age. First, a holistic and socio-ecological view of reintegration is needed [35]. This means acknowledging multiple areas of need across all areas of life and wellbeing, as well as the multiple levels of influence on a person’s reintegration outcomes. Studies have consistently demonstrated a breadth of needs for older people after leaving prison. Qualitative studies from multiple stakeholder perspectives (e.g., parole officers, caregivers, transition support services and prison leavers) paint a grim picture of a highly challenging time with intense support needs that span almost all areas of health and functioning; Jimenez and colleagues capture the story well in their paper title: “He needed just about everything…” [36]. Common challenges are managing multiple physical, mental and cognitive comorbidities, lost social connections with age, (in)continuity of care, stigma and difficulty accessing housing, high dependence on caregivers, a lack of confidence and fear of post-release life [22–24,26,28,37–39]. Quantitative research similarly demonstrates high healthcare needs, risk of mortality and homelessness [40–43]. The arising issues call for solutions that involve multiple sectors across health, justice and social services, and the general community, as well as change at multiple levels (individual, organisational and government/societal) of these sectors. Successful reintegration of this aging population is a necessarily transdisciplinary subject, thus, we approached the qualitative analysis mindful of a range of criminological, social work and public health concepts.

The second and related consideration is having a transdisciplinary focus on reintegration or adjustment in the community, rather than desistance, which is a primarily criminological perspective. Desistance centers on the ceasing of criminal behaviour, whilst reintegration has a broader focus on adjustment. Recidivism is an important outcome to consider in reintegration and is inevitably of high interest to policymakers. However, desistance-focused approaches can be limiting. Desistance from crime and associated criminogenic needs that often underpin release interventions are inextricably linked to broader adjustment and functioning across life [44]. Directing attention toward reintegration fosters more expansive and lasting effects, encompassing both the cessation of undesirable behaviors and holistic well-being. Studies with formerly incarcerated individuals also advocate for conceptualizing life after prison as one that goes beyond “not offending” [45]. This approach generates positive results not only for the justice system but also for several sectors that would otherwise bear the consequences of unsuccessful reintegration in this growing population in the community, such as public health, aged care and social services.

Finally, we agree with the increasingly prominent opinion that reintegration efforts targeted at those who are justice-involved should be strengths-based and solution-focused. There has been widespread advocacy and evidence for strengths-based approaches to replace the traditionally deficit-based and risk-management approaches to rehabilitation that find their roots in the medical model [46]. Leveraging capacity rather than emphasizing risk is an obvious choice where the motivation and cooperation of the individual themselves is critical. In a review of desistance efforts, Fox concludes that “Interventions that have the greatest success tend to be the ones that emphasize the strengths of the individual or help to enhance the more pro-social aspects of their selves.” [47]. In summary, this study aimed to construct a novel conceptual framework to understand release and reintegration for people leaving prison in older age, with a holistic and strengths-based focus on enablers for reintegration.

## Methods

### Ethical approval

This study was granted ethical approval from: The University of New South Wales Human Research

Ethics Committee [HC220042], Corrective Services NSW Ethics Committee [D2022/0294030], and

the Justice Health and Forensic Mental Health Network of NSW Ethics Committee [G477/22].

### Qualitative approach

This study used Charmaz’ constructivist approach to Grounded Theory, which seeks to construct an explanatory theory or conceptual understanding about a phenomenon [48] using constant comparison. It “encompasses the interconnections of concepts and categories to interpret and explain patterns or process(es) of a psychosocial phenomenon” (Charmaz, 2014), employing a mix of inductive (inferring from the observed data), deductive (inferring by testing against existing theories), and abductive reasoning (inferring by creatively ‘filling in the gaps’) to analysis.

Charmaz’ approach was most suited for this study for three reasons: it lends itself more to practical application than other approaches, aligns better with highly subjective and personal accounts of a complex social phenomenon, and harnesses the researchers’ expertise and existing literature to construct the theory [49]. Charmaz’ constructivist-interpretivist approach rests on a relativist ontology and subjective/interpretivist epistemology. This means that rather than seeking to find an objective reality that exists ‘out there’, this approach believes in the existence of multiple perspectives of reality, depending on the subjective experience and local context of each person. It also acknowledges the researcher as a highly involved, co-constructor. It values the professional and sometimes personal experience of the researcher as something that cannot be separated from the theory construction process and takes advantage of their knowledge of literature and intuition, rather than limiting the theory to be created from just the data at hand. Charmaz’ approach was also suitable for our intent to highlight enablers/intervention points. The study design and analysis were interrogated to align with the eight key markers of qualitative quality espoused by Tracy [50]: worthy topic, rich rigor, sincerity, credibility, resonance, significant contribution, ethics and meaningful coherence.

In addition to this approach, we specifically aimed for development of a conceptual framework, as defined by Jabareen et al [34]. A conceptual framework can be described as an interconnected set of ideas that together provide a comprehensive explanation of a phenomenon. Importantly, the individual concepts within the framework support one another, clarify the specific aspects they represent, and collectively establish a distinctive guiding philosophy unique to that framework. A conceptual framework, whilst not a fully elaborated theory, is a suitable pragmatic application of Grounded Theory [51] and ideal for preliminary explorations of phenomena due to its flexible, and modifiable nature, as well as our emphasis on understanding, rather than prediction [34].

### Data collection

Data was taken from two main sources. First, semi-structured interviews were undertaken with individuals who have lived experience of being released from prison in older age. Inclusion criteria were:

- Released from an Australian correctional centre (prison) in the past 24 months
- Aged 50 years or older at release (45+ if an Aboriginal or Torres Strait Islander person)
- Spent at least 12 months incarcerated (sentenced or on remand)
- English ability sufficient to participate in a 60-minute phone interview

These interviews were undertaken as part of a broader study which aimed to investigate and improve post-release outcomes for older people leaving prison in Australia. There were three sections to this interview. The first section informed the current study and consisted of open-ended questions about their experience of release from prison and reintegration in older age. Participants were asked to describe preparation for release, their experiences and circumstances after release, what the key challenges and supports were, and what was important for their reintegration. The other two sections focused more specifically on experiences of health management and technology use after release from prison, as these had been identified as pertinent issues for this population [33,52]. These sections were not the focus of this study, but inevitably informed the researchers’ knowledge as per the constructivist grounded theory paradigm discussed above.

Recruitment occurred via community-based organisations who provide services to individuals who have been released from prison in Australia, and ‘community corrections’ offices throughout New South Wales, Australia, who are responsible for the supervision of individuals who are serving community-based sentences or are released on parole. Recruitment occurred from 01/05/2022–31/08/2022. Verbal consent was obtained prior to the interview using a verbal consent script and audio recording, as approved via ethical review. Details regarding the consent (date, time, verbal nature, participant’s name) were recorded in a spreadsheet, and the consent itself was audio-recorded. These records were hosted on password-protected folders on the university’s shared drive. Phone interviews were conducted by two researchers, each interview took 30–60 minutes and participants were renumerated 75AUD for their time.

The second source of data was a series of online workshops which aimed to identify issues pertinent to reintegration of people leaving prison after middle age (50+). Participants included purposively sampled staff and stakeholders with at least 12 months professional experience supporting or conducting research on older people leaving prison. Key organizations and services with the most relevant knowledge were identified and contacted through publicly available contact details via email or phone. These included post-release transition support services, aged care providers, advocacy groups, parole officers, and prison health staff. A member of the research team explained the study, sent study information, and sought assistance in identifying potential participants. Staff within each organization agreed to distribute relevant study materials to potential participants. Potential participants were individually invited to the study through an email invitation that included a Participant Information Statement and Consent Form, and a study flyer. Participants could express interest by contacting the research team via email or telephone. Once interested, the research team conducted an eligibility screen using the inclusion and exclusion criteria. Eligible participants received an online version of the consent form to complete by clicking relevant checkboxes. Recruitment across groups occurred simultaneously until a maximum of four participants from each stakeholder group was identified. Staff who were under the age of 18 or whose relevant experience ended more than five years prior to the study were excluded.

The workshops were run by one facilitator and one research assistant. First, the facilitator presented a brief review of available evidence regarding older people in prison and post-release outcomes. Then, participants were invited into open discussions of what they have experienced to be key issues defining the experiences of people leaving prison at an older age, current strengths and opportunities to improve outcomes during this time, and what ideal reintegration would look like. Recruitment for the study consisted of contacting relevant organisations or services via publicly available details and using existing contacts of the research team. Recruitment occurred from 01/05/2022–31/08/2022. Written consent was obtained prior to the study via an online form. The workshops ran for 90 minutes each and participants were renumerated 50AUD for their participation.

### Participants

The total sample size for this study was N = 30. Demographic characteristics of the fifteen lived experience participants are presented in Table 1.

**Table 1:** Demographic characteristics of lived experience interview participants (N = 15).

Demographic characteristic	*n* (%)
Mean Age (SD; Range)	57 (6.3; 47-69)
Male	14 (93%)
Aboriginal or Torres Strait Islander	6 (40%)
Born in Australia	15 (100%)
Mean months since leaving prison (Median, Range)	8 (6, 1-24)
Most recent state of imprisonment	
- New South Wales	12 (80%)
- Victoria	3 (20%)
Highest education	
- No qualification	4 (27%)
- Year 10	7 (47%)
- Diploma	1 (7%)
- TAFE or trade certificate	2 (13%)
- Postgraduate degree	1 (7%)
Living Situation	
- Own home or with family	7 (47%)
- Public housing	6 (40%)
- Renting privately	2 (13%)
Current income source^a^	
- Government payment	14 (93%)
- Full time work	1 (7%)
- Part time work	1 (7%)
Times in prison over lifetime	
- Once	4 (27%)
- 2–5 times	6 (40%)
- Over 10 times	3 (20%)
- Undisclosed	2 (13%)

^a^One participant reported both government payment and part time work

Staff participants included prison health providers (n = 3), corrective services staff (n = 3), post-release transition support services staff (n = 4), researchers (n = 3) and staff from prison and older age advocacy groups (n = 2). Whilst most stakeholders were currently employed in New South Wales, many had professional experience across multiple Australian jurisdictions.

### Analysis and framework development

**Fig 1:**
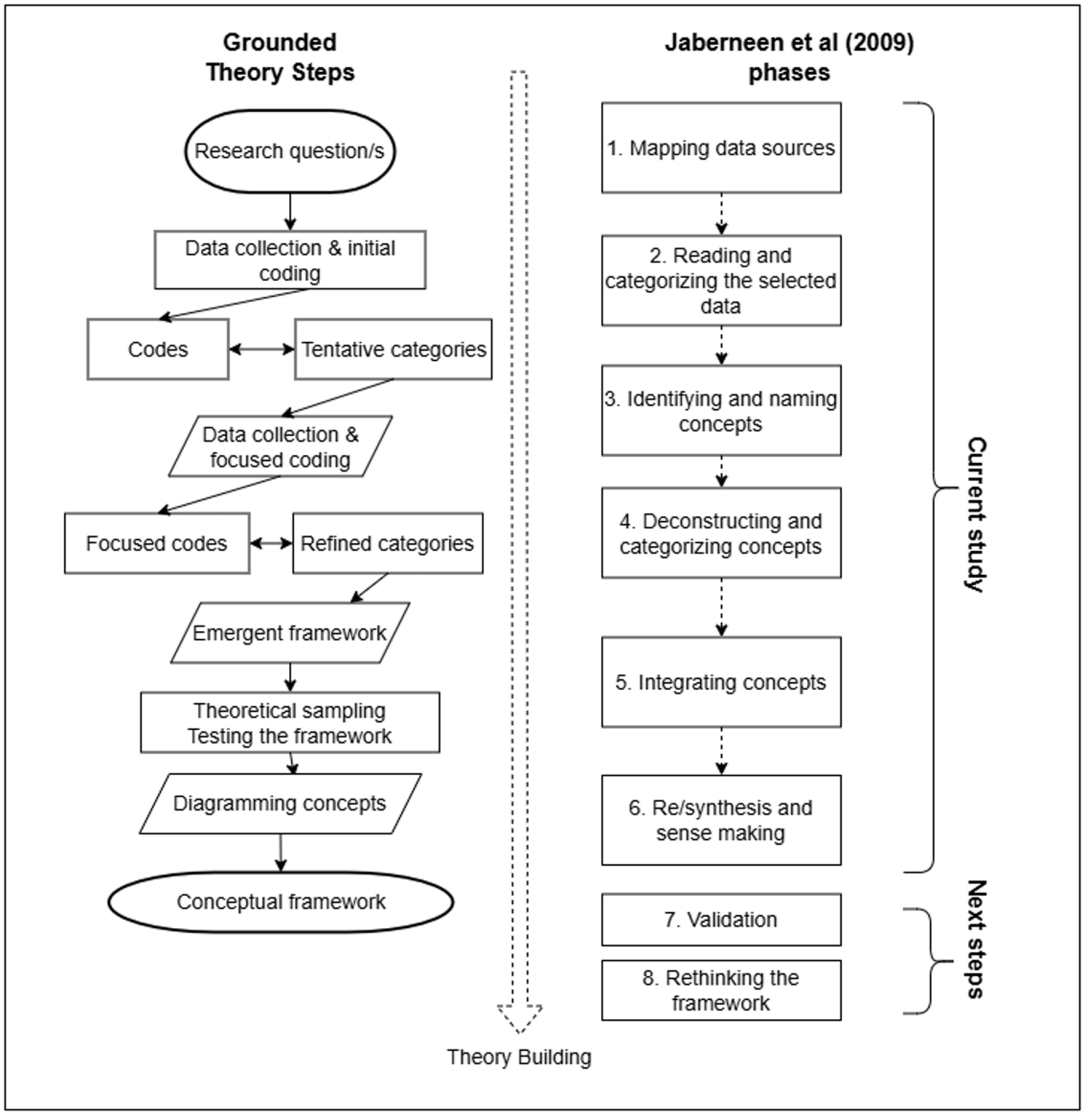
Grounded Theory process for conceptual framework development alongside relevant phases described by Jaberneen et al (2009).

Fig 1 presents the steps involved in grounded theory that were followed in this study, alongside the eight concurrent phases of conceptual framework development proposed by Jabareen et al [34]. The analysis followed Charmaz’s (2006) constructivist grounded theory approach, which employs iterative coding, constant comparison, and abductive reasoning to construct a conceptual framework. We distinguished between concepts (theoretical constructs derived from codes) and integrated concepts (synthesized higher-order constructs). This terminology reflects Jabareen’s (2009) emphasis on building a network of interconnected theoretical ideas rather than identifying thematic patterns alone.

Interviews and workshops were audio-recorded, transcribed and imported to NVIVO12 software for analysis by a research assistant. The interview transcripts were subject to the creation, development and refinement of codes (organising meaningful sections of data) and memos (notes reflecting ideas from the data and researchers’ knowledge/experience). Related codes were pulled together into categories. These were further grouped into underlying concepts at a broader level (subthemes). These concepts underwent further integration and synthesis into higher level themes, and ultimately temporal stages. These stages emerged to represent how the high-level themes fit together into a framework that is useful for describing the strengths-based reintegration of a person after release from prison. Pertinent quotes from each stage were chosen for presentation alongside the results.Overall, an iterative process of testing and refining the emerging framework was undertaken throughout, as well as revisiting literature, the parameters of the framework outlined in the introduction (solution-focused, transdisciplinary, holistic reintegration) and the original collected data, until a final framework was developed.

## Results

### Framework overview

**Fig 2:**
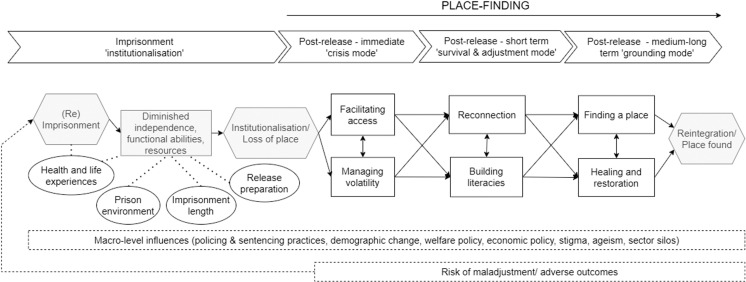
Final simplified conceptual framework for the ‘Place-finding Framework of Reintegration’.

Table 2 presents the integrated concepts (themes), concepts (sub-themes) and categories (codes) for each stage, and Fig 2 is a representation of the emergent framework overall. The framework can be broken up into four stages, following an individual from institutionalisation to reintegration. The overall process of reintegration can be conceptualised as a journey of ‘place-finding’ (global theme). The framework also acknowledges the influence of macro-level contexts (e.g., legal, political, cultural and economic) on outcomes at each stage, as well as an ever-present risk of returning to prison.

**Table 2:** Key components of framework development presented by stage.

Stage	Integrated concepts (themes)	Concepts (sub-themes)	Categories (codes)
Institutionalisation	(Re)Imprisonment Diminished independence, functional abilities, resources	Length of imprisonment Health & Life Experiences Prison environment Release planning	Education Trauma Previous imprisonment Mental, physical and cognitive health Socioeconomic status Health & functional declines Limited independence • Social disconnection & segregation Restricted access to information and communication technology Limited or untailored release preparation Risk management focus Service/sector silos
Crisis mode	Facilitating access Managing volatility	Crisis needs Care continuity Intense support	Identification documents Financial assistance Immediate, age-appropriate housing Technology access and literacy Drug & alcohol support Medication Medical record sharing Health equipment Integrated care Case management Wraparound care Supportive relationships Psychoemotional resilience Motivation Resilience Help-seeking Feeling prepared for release
Survival & adjustment mode	Reconnection Building literacies	Managing daily life Social reconnection Learning	Learning – key literacies Increasing self-efficacy Social reconnection Overcoming stigma Motivation to desist Actively managing health Adjusting to daily activities Stable and appropriate housing
Grounding mode	Finding a place Healing & restoration	Establishing long-term patterns Negotiating positive identity Contribution	Productive activity Social participation Long-term housing Established, independent daily routines Long-term outlook Supportive social networks Health-seeking behaviours and health management Healing from trauma Restorative justice Parole fulfillment Positive self-identity and self-esteem Shedding criminal identity
N/A	Macro-level influences	Culture Policy Demographic change	Policing & sentencing Demographic changes Policy – welfare, economic Stigma Ageism Sector silos

### Stages

**Fig 3:**
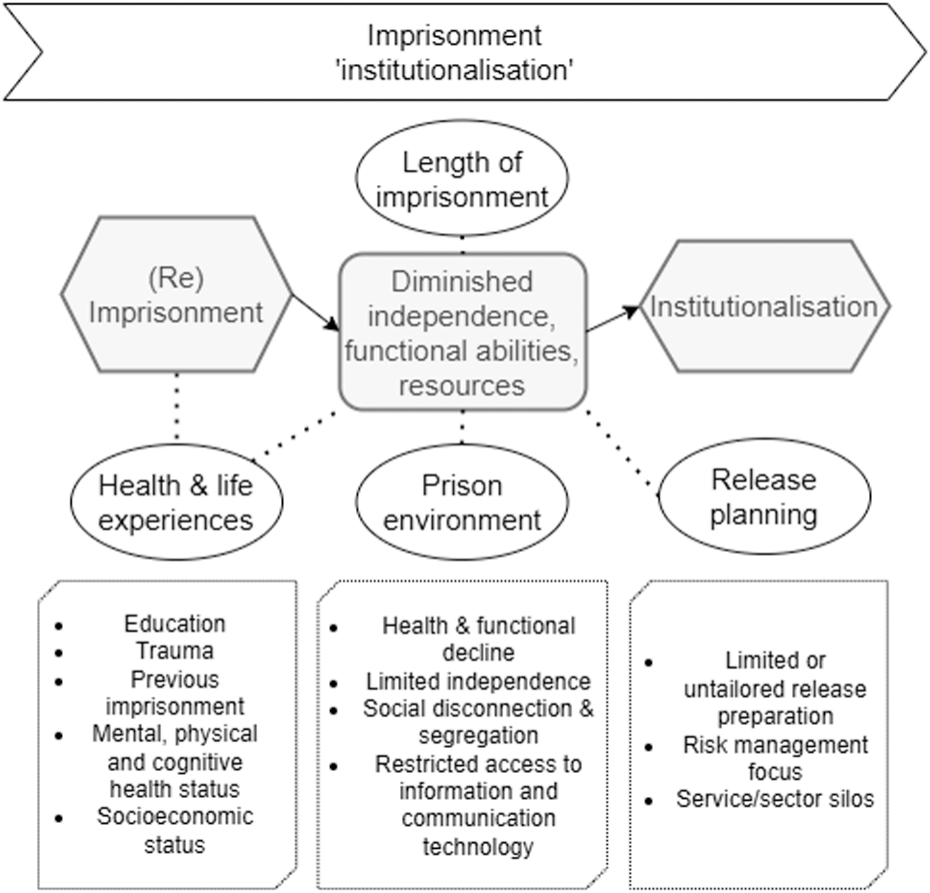
Expanded illustration of Stage 1: ‘Institutionalisation’.

**Fig 4:**
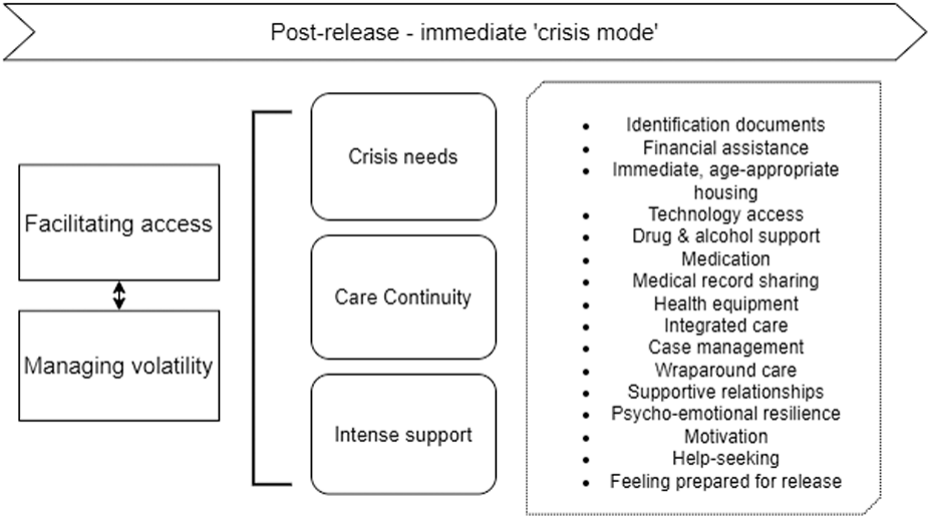
Expanded illustration of Stage 2: ‘Crisis mode’.

**Fig 5:**
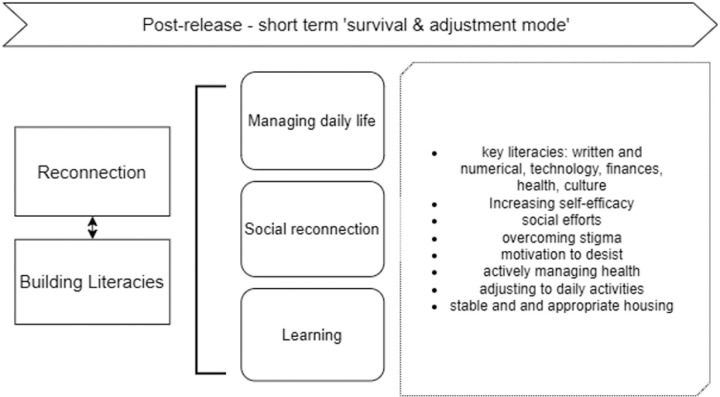
Expanded illustration of Stage 3: ‘Survival and adjustment mode’.

**Fig 6:**
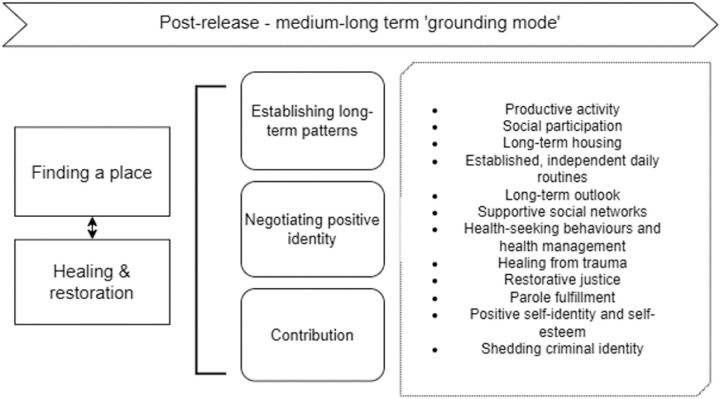
Expanded illustration of Stage 4: ‘Grounding mode’.

Reintegration can be considered a process of ‘Place-finding’, across key temporal ‘stages’: ‘institutionalisation’, ‘crisis mode’, ‘survival & adjustment mode’, and ‘grounding mode’. Each stage has its own unique focus. The stages are not just illustrative of a timeline of events, but argue for the consideration of the right influences, supports and priorities, at the right time, to enable successful reintegration. The temporal boundaries between the stages are not fixed or uniform; rather, the time spent in each stage, as well as the specific challenges and needs encountered, can vary between individuals. Figs 3–6 presents expanded illustrations of each stage. Participant quotes are identified using codes (e.g., P1, P2, P3) for lived experience participants and (e.g., S1, S2) for staff or stakeholder participants.

#### Stage 1: Institutionalisation (Fig 3).

Prison is described as a restricted and regimented setting that is segregated physically and socially from the rest of the community. Periods of incarceration inevitably undermine a person’s ability to perform relevant and up to date societal functions and their independent functioning. As one individual described, incarceration eroded their knowledge of societal norms post-release:

*“It’s very hard when you get kicked out and then put in the community, to know everything… because it’s all different now. ” – P4*

Stakeholders further noted that prolonged imprisonment exacerbates this disconnection:

*“They’re leaving after five years, and it’s quite a secure environment for them. And then going out to the big world.” – S1*

Increased time in custody also corresponds to deterioration of social support in the community.

*“There are also family members who you know… disown them” – S2*

This effect can be perpetuated via lengthy or repeated experiences of correctional detention from adolescence through to adulthood, and in other similarly restrictive care settings. Simultaneously, inmate populations tend to experience poor social determinants and a range of health challenges across physical, mental and cognitive domains. Prisons are primarily places of detention and security, and resultantly ill-equipped to provide care and prevent health and functional decline in inmates.

*“Things like is the functional enablement and maintaining function that it’s left to people’s own devices.” – S3*

Over time, an individual diminishes in their support networks and capacity for self-care and independent functioning in the community, whilst their need for more and more complex forms of support increases. We refer to this process as ‘institutionalisation’. One participant reflected on the loss of structure post-release:

*“For better or worse, jail does provide you with a routine, whether you like it or not, you do adjust to living to quite a strict regime of routine in prison. And of course, when you get out, that’s gone.” – P3*

The intervention focus during this stage should thus be on preventative care and maintaining functional abilities whilst in prison, preserving social supports available to the person in the community, and equipping and updating individuals’ capacity for independent and societally relevant functioning after release. A strong focus on release preparation is clearly needed, as a participant reflected:

*“It’d be better if they taught you all this before you get out. Like you know what I mean? not just talk when your release date comes up, letting you out, then you gotta figure it out all by yourself” – P2*

In particular, there was expressed concern that existing release preparation was narrowly focused on reducing the risk of reoffending only.

*“All the courses that they have for you to do are all to do with your offending” – P15*

#### Stage 2: Crisis mode – facilitating access and managing volatility (Fig 4).

This period refers to the immediate period after release from prison, often described as *“the first days out”,* and is characterized by high volatility in the individual’s life emotionally, mentally, physically, socially and financially. Individuals reflected on the abrupt transition where they were often without key resources needed for immediate life in the community:

*“I’d lost me home, so I lost everything. I’m getting out to nothing, you know?”- P5*

The two focuses of this stage are thus to ‘facilitate access’ to urgent needs and to ‘manage volatility’. Enabling access to the most urgent health, social and welfare support takes precedence during this stage. Priority areas identified in this stage included immediate access to post-release housing and finances, access to internet and mobile phones, personal identification documents and medical records, as well as immediate health needs such as medication, medical devices and mental health or substance use support. Participants highlighted gaps in preparing individuals for release such as medication and documentation:

*“You know, people are released from prison and they haven’t gotten proper ID and you know, you can’t be released into the community unless all those things are set in place for you.” – P1*

*“You only get one week’s medication on release, and that simply is not enough.” – S4*

Participants reflected on psychoemotional resilience, motivation for successful reintegration and their ability to seek help, as being key for managing volatility:

*“When you get released, you’ve got to have that confidence. You got to have that confidence to build, courage to sort things out.” – P4*

*“When you’re younger all you wanted to do was get out and hit drugs go back to the same old sort of thing, but my attitude was different. Yeah. I just thought this time I’m gonna make it this time, you know?” – P9*

The critical role of transition support during the immediate post-release period was often emphasized. Both formal case management services and informal caregivers were described as instrumental in helping individuals navigate the complexities of re-entry, particularly when support was intensive and tailored to individual needs. Effective support involved not only facilitating access to essential services and resources, but also providing practical assistance with daily tasks and reminders, which helped participants manage the significant changes they faced after release. For example, one participant explained how their caseworker provided ongoing organizational support:

*“[caseworker] always organised stuff for me. I signed on to do things and I can’t follow through, but she will. She’ll remind me I’ve got an appointment. She’ll remind me to do things you know.” – P8*

Another participant described the sense of security and stability that intensive case management offered in a period of vulnerability:

*“When I was released from jail, and I was bit scared... I felt so secure after about two weeks being with [the caseworker]… [She] virtually got me everything. A phone, clothes. If it wasn’t for her, I wouldn’t be here today. I probably would have relapsed and been back in jail… [she’s] been a mentor in my survival, really.” – P7*

#### Stage 3: Survival & adjustment mode – scaffolding supports and building literacies (Fig 5).

Survival mode is characterized by less acute needs compared to crisis mode, and an increasing focus on reconnecting with society and building key literacies that will form the foundations for an adjusted and settled life in the community. Important features of this stage include social reconnection, often related to overcoming stigma about their imprisonment history, having acute health and substance use issues under control, gaining independence and confidence in daily activities, and actively managing longer term physical and mental health needs. For example, one participant shared how initial feelings of anxiety and reluctance to engage with others gradually gave way to renewed social connections:

*“I was just not wanting to go anywhere or do anything because … it was kind of a paranoid feeling. And it wasn’t probably until I have been out for three weeks that I rang the guy that was the pastor of my church and said, look, I’d like to come to church. And he said, ‘Yeah, come, come. There won’t be any problems there.’ So I started there, and then I started going for a few walks and slowly, slowly back into all that.” – P3*

Rebuilding family ties was also a common theme, with participants highlighting the importance of making consistent efforts to reconnect:

*“I wanted to build that relationship back up with my kids… after some time I’d make an effort every weekend. Together, the Sunday lunches at mum's and that.” – P8*

The overall concept of ‘learning’ or ‘adjusting’ is pertinent to this stage and individuals build literacies in key areas of daily life such as housework and financial management. Many spoke of having to start over with basic tasks, such as managing finances or maintaining a home, which could feel daunting after long periods of institutionalisation. As one participant explained,

*“Yeah I had to learn things, and start all over again. So many can’t manage money and even learning to pay your own rent can be daunting for people…. So just basic skills like that.” – P9*

Adjusting to the rhythms of everyday life also took time, with some noting the challenge of re-establishing routines:

*“It was a bit hard getting used to the just the cooking, sort of use of the time used to the normal life again I suppose it took probably two months for me really? Till I got back in the real world, if that makes sense.” – P14*

Low education, especially written and numerical illiteracy, pose a common challenge for acquiring key skills such as technology or accessing healthcare, and restrict confidence.

*“I upload things and send emails now, which is cool. It took me a couple months to learn, I’m a slow learner I’ve always been a slow learner.” – P6*

Several participants identified age-related barriers to acquiring essential skills, particularly digital literacy. As one individual explained,

*“I’m bloody hopeless at using tablets and computers and stuff. But I know now, I’ve got to learn, because if i’m going to survive in this world. At my age, I’ve got to learn how to use them. It’ll take a long time for me to work things out, because I’ve got a thick head, and it takes a lot to go into this thick head of mine.” – P4*

Support needs remain very high at this stage, and building ongoing supportive social connections are important to achieve literacy in areas where they remain lacking in skill and/or confidence. Transition to this stage from ‘crisis mode’ required the individual to have had their immediate health and safety needs met:

*“I couldn’t get myself out of my house to go to programmes because firstly, I’d get scared, because my phobia I don’t want to be around people. And secondly, I’d rather get over the hurdle of mental health problem before I even go to trying to learn something new. I’m just trying to maintain my.. just I don’t want to [drug] relapse, don’t I don’t want to fall back. I’d rather just get stable with the home. Once I sort them issues out I think everything will be, I’ll move forward. definitely.” – P8*

#### Stage 4: Grounding mode – healing and finding a place (Fig 6).

During the grounding stage, participants described a shift from focusing on immediate survival needs to establishing longer-term stability and a renewed sense of self. Many spoke about the process of healing from past negative experiences-including trauma that predated imprisonment-and how this was essential for moving forward. For example, one participant reflected on the impact of psychological support in addressing longstanding issues:

*“And on Saturday I go and see the psych[ologist], which really helps, because I’m going through the [childhood trauma] stuff. I haven’t lived, I’ve existed, for the majority of my life. But not anymore.” -P7*

A key marker of reaching this stage was the development of new routines and a sense of stability in daily life. This included maintaining regular healthcare appointments, establishing healthy habits, and securing stable housing. For instance, participants emphasized the importance of having a home environment that felt safe and under their control:

*“I have a place to live. And to have a quiet area, you know, to live in and to forget about having been inside [prison].” -P4*

*“I’ve just set my place up the way I like it.” – P1*

Others described how developing daily routines, such as regular exercise or managing finances, contributed to a sense of normalcy and accomplishment:

*“Whenever I want to go out, I’ll make sure I stay out for 25 to 30 minute walk. And I try and do that twice a day if it is not raining.” – P3*

*“I’ve got over 2000 dollars saved already and next Friday I’ll have 2300 or something saved. I get my [driver’s] licence back on the ninth, and then I’m going up [interstate] to visit my daughter who I hadn’t seen since she was two. Yeah. So I’ll spend some time with them.” – P7*

Confident participation in community life was another indicator of grounding. Some participants described reconnecting with local institutions and social groups, which helped foster a sense of belonging and identity beyond their prison experience. As one participant shared:

*“I’ve pretty much been adopted by the local library as a life member because I am down here a lot using the computers.” – P3*

In addition, many found meaning and a positive sense of identity through contributing to society-whether via restorative justice activities, volunteering, or employment. These experiences were seen as important steps in negotiating a new place in the community and letting go of the negative aspects of the past.

### Macro-level influences

Many broader macro-level influences affect the place-finding journey for individuals (Fig 2). Those at play during institutionalisation mostly stem from the institutional context of prison settings, i.e., sentencing laws, policies, demographics, resource allocation and sociopolitical perspectives regarding how prisons should operate. For example, a stakeholder observed that prisons primarily focus on managing criminogenic risk rather than addressing individuals’ underlying problems:

*“Prison isn’t a place where you deal with your problems. It’s all focused on risk. Criminogenic risk.” – S5*

Once released, the socioeconomic and political landscape will continue to shape the individual’s access to resources such as post-release welfare support. Stakeholders described how minimal post-release support often leaves individuals vulnerable:

*“Someone who’s released from custody doesn’t have any supports, is given two nights of accommodation and is given basically the release papers. And that’s it. – S6*

Also influential are policy and cultures around siloed service delivery across justice, health, and community sectors. Participants spoke about the fragmented nature of service delivery across justice, health, and community sectors, which can hinder continuity of care:

*“Once you are out the front door, you will no longer the department of justice’s problem” – S7*

Similarly, ageism and the lack of age-appropriate policies can significantly impact access to safe and equitable healthcare, housing, employment or socialization opportunities. Several participants shared experiences of being underestimated or marginalized due to their age:

*“People talk to you like you’re dumb and stupid because you’re 60 and they think you know everything. And I’m still learning about life and i’m still learning how to work things.” – P2*

*“Whereas the young one, you know if they don’t go back to prison, I mean, they can do jobs that probably women over 50 and 55 can’t do. We are really restricted.” – P1*

A strong persisting challenge across stages is the stigma associated with being a person with a criminal history. One participant expressed frustration with community attitudes:

*“The community don’t really grasp the notion of do the crime, do the time and move on, unfortunately.” – P3*

Similarly, stakeholders noted that stigma can affect access to aged care services:

*“Aged care organizations that are reluctant to, you know, accept older prisoners mainly because of their crime history and stigma around that.” – S1*

### Bringing it all together: “Place-finding” after disconnection

When considered together, the framework supports ‘place-finding’ as useful concept to understand a person’s journey from institutionalisation to reintegration (Fig 2). That is, a person loses their place in society as a result of the institutionalising and disconnecting processes inherent to incarceration. The extent of this loss of place will vary for individuals, and are the result of a mix of factors across pre-imprisonment life, imprisonment experiences, and the prison setting itself. When people are released, they embark on a journey of place-finding. The priorities immediately upon release (crisis mode) are on the resources needed for ‘landing safely’ in society, after which individuals begin processes of reconnecting with their daily lives and learning to adapt to their social and physical surroundings (survival and adjustment mode). Finally, in the medium-long term (grounding mode), individuals negotiate their long-term identities in society. The process of place-finding is supported or hindered by key enablers both at the micro (individual) and meso (society, organisations) level, whilst understanding influence and advocating for changes at the macro (national and global) level. The unique support needs that an individual experiences as part of their place-finding within each stage are uniquely shaped by their life experiences, personal resources and socio-cultural contexts.

## Discussion

### Overview

The ‘Place-finding’ framework for reintegration offers a useful, preliminary way of understanding the emerging literature regarding leaving prison in later life. The framework provides a way to better understand the challenges and outcomes experienced during reintegration, as well as stage-specific targets for intervention. The framework is holistic and socio-ecological, acknowledging that reintegration is not solely determined by individual efforts, but by the dynamic interactions with the contexts and systems surrounding the individual. Moreover, although rooted in the criminological concept of reintegration, the workings of the framework are transdisciplinary, incorporating insights from psychology, social work, and public health to capture the complex and multifaceted nature of reintegration as a ‘whole of life’ process. Resultantly, interventions can and should be shaped by considering and integrating multidisciplinary approaches.

The framework reveals the unique and complex needs that arise for older people in the context of imprisonment and release, highlighting issues such as chronic health conditions and reduced social and economic capital. For example, participants described difficulty adapting to new technology, a challenge that reflects age-related digital exclusion, as well as research that highlights the digital divide as being particularly acute for older prison leavers, compounding their risk of social isolation and exclusion. Moreover, the recurrence of health issues to be attended to at each stage reflects health decline is often accelerated in older prisoners due to the combined effects of ageing, pre-existing health issues, and the prison environment, which is not well equipped to support age-related needs. Similarly, social support is a recurring issue, the lack of which is exacerbated by age, as older individuals are more likely to have lost contact with family and friends during long sentences, and face additional stigma related to both age and criminal history.

However, despite being grounded in data regarding older people leaving prison, this conceptual framework accounts well for individual variability and is adaptable to people leaving prison or other similar institutional settings at various ages. This is because the framework in its simplest form posits that any incarceration experience and subsequent loss of capacity, resources and networks, leads to a state of institutionalisation that varies for each person. The challenges and needs at each stage remain relevant challenges to others who have experienced incarceration, with specific priorities being substitutable depending on the individual’s circumstances. Notwithstanding this, the authors acknowledge that the framework in its current state best reflects the reintegration of those who have experienced lengthy or repeated periods of incarceration and are released after middle age.

In a similar vein, the framework can be applied and tested in various institutional settings. According to this framework, prisons that share similarities in how segregated their occupants are from society and how much they invest in preserving or replenishing societal functioning and resources in preparation for release, will inevitably release people who look similar in terms of their release challenges and needs. This can explain why, despite the geographical distance and/or varying governance across prisons, studies across English-speaking countries who also share historical and cultural underpinnings in law and penal systems tend to converge on similar post-release challenges.

### Contribution to literature and theory

Overall, the main issues for reintegration identified at each stage complement the post-release challenges and needs for older people reported in other studies – e.g., housing instability, agency and empowerment, high and complex care needs, service access challenges, social isolation, stigma [31,32,53–55]. Further, this conceptual framework ties all these factors together, grouping and identifying key underlying stages to delineate and explain what is being observed across both qualitative and quantitative studies more clearly. It thus provides more explanatory power than such studies, and facilitates the design of targeted interventions and supports, delivered at the right time. For example, in their qualitative work investigating the experiences of caregivers for people leaving prison in older age, Jimenez et al (2021) also identified “developing self-sufficiency” as one of three main themes, revealing challenges in “scheduling tasks and forgetfulness”, “applying for benefits and services”, and “adapting to new technology”. They highlight the challenge of balancing direct support provision for these individuals, whilst enabling them to complete these tasks themselves, and additional concerns about the caregivers themselves aging and losing capacity to provide care. In response, the current framework would see to the prioritization of direct support provision in initial stages (crisis mode), followed by a transition towards scaffolded support to empower individuals in managing daily schedules and enhancing their technology literacy (survival & adjustment mode), with a view to maximizing independence in the long term (grounding mode).

From a social work standpoint, the framework complements systems theory in it placing the experience of the individual within complex and interconnected systems, and additionally the many challenges that are faced at the intersection of moving between systems. Accordingly, the socio-ecological model [35] is also aligned in highlighting the unique experiences of living within, and being released from, the institutionalising prison environment (mesosystem), which are designed and run by the sociopolitical structures of the broader world (exo- and macrosystems). Moreover, the strong influence of personal resources and social supports on this experience (micro- and mesosystems).

The framework maps well to key theoretical concepts and practice models across existing social work and criminology. In terms of desistance theory in criminology, topics covered in the framework include: agency and personal transformation, social bonds and support, turning points and life events, cognitive and emotional changes, and rehabilitation-focused intervention. The idea of a staged approach or the passage of time is also not a new concept in existing desistance literature [47,56]. However, compared to existing work, our framework argues for attention on briefer periods of time and a focus on the immediate post-release period, as well as the critical time-dependence of needs and intervention during each period. Our model also supports a larger focus on meeting health needs, as proposed by Link et al. [57], and additionally illustrates the process of cumulative health decline and loss of health-related self-efficacy and literacy during imprisonment that leads to this being a key reintegration issue.

A unique theoretical contribution made in this study is the identification of a cumulative, ‘institutionalising’ effect of imprisonment as a key mechanism involved in subsequent reintegration prospects. Reintegration is essentially a reversal of the disconnection and incapacitation that occurs during imprisonment. In this way it encourages recognition of imprisonment as a fundamental part of the reintegration process, and explains why lengthy, repeated incarceration can be particularly harmful.

Regarding more practical models of rehabilitation, the framework is mostly compatible with the Risk Needs Responsivity (RNR) [58] and Good Lives Models (GLM) [59] that are currently popular. It shares values from both models such as individualized approaches, targeted interventions and a holistic perspective of a persons’ needs. Regarding the RNR model, the ‘responsivity’ principle offers a practical way to design interventions at each stage of our framework and is compatible in its focus on cognitive-behavioural-based change and consideration of both internal and external influences in shaping responses to interventions. Meanwhile, our framework further highlights the existence of a highly volatile ‘crisis mode’ stage, in which responsivity will likely be impaired.

A point of departure from the RNR model is the strengths-based approach of our framework, which is concerned primarily with enabling factors, rather than criminogenic risk, as the central factor upon which solutions should be designed and allocated. However, this does not mean the two are incompatible. The most considered risk/need factors that are used by the RNR (i.e., criminal history, pro-criminal attitudes, pro-criminal associates, anti-social personality pattern, family/marital status, school/work, substance abuse, leisure/recreation) do complement the reintegration objectives of our model. It is also still possible for interventions from the RNR to be developed with a strengths-based approach, to enable people to address these risks. However, our framework encourages a closer look at the underlying enabling processes to attain these outcomes, and the longer-term value of equipping a person with relevant efficacies. In line with this, the framework puts greater emphasis on the ‘non-criminogenic’ factors believed by the RNR as being indirectly related to risk, such as self-esteem, trauma and physical health. Our framework also extends the horizons of RNR-based interventions, considering the ultimate goal of reintegration as achieving equivalent adjustment and functioning to those in the general community, rather than simply avoiding reoffending.

The GLM is arguably more aligned to the model in its strengths-based approach and particularly its focus on building individual capacity, with the end goal of ‘healthy human functioning’. One reason that the framework may align more strongly with the GLM than the RNR is that it was built on qualitative consultation with stakeholders, rather than quantitative data. The GLM has notable human rights underpinnings, which are more likely to be advocated for by participants of qualitative research, compared to the RNR which relies more strongly on quantitative evidence regarding predictors of recidivism.

For both the RNR and GLM, the Place-finding framework offers stronger theoretical bases in terms of causal timelines, critical stages and the idea of deinstitutionalisation, to help make sense of, and better focus, interventions that work. Unlike both models, it highlights imprisonment experiences as a core factor in reintegration. This is a crucial finding, because it forces onus on correctional settings to consider their environments in reducing recidivism and enabling successful reintegration.

### Implications for practice

The principles of this framework can be applied to inform services and interventions that are delivered to this population. That is, they can be mapped onto the priority areas relevant to the person’s reintegration stage. They should be developed with a long-term perspective that aims for equivalent levels of health and functioning to those in the general community who have not been incarcerated and recognize that this is achieved through a process of ‘deinstitutionalisation’ or ‘place-finding’.

The framework encourages time- or stage-based thinking. That is, it calls for interventions to be designed and prioritised based on which stage an individual is experiencing. This also means that an individual’s current state or outcomes can be better understood by looking back to experiences in previous stages. Maximizing timely support delivery during each stage will ensure best possible outcomes and prevent return to prison. Using these guiding principles, the framework can also be used to evaluate whether or why past, existing or planned programs have worked for this population.

There are two parts to the implementation of these findings: the responsibility of prisons to maintain a person’s place in society while a person is incarcerated, and the availability of timely and sensitive health and social services in the community once a person is released to ensure they can re-create or newly establish their place in society. First, there is a greater need for prisons to accept responsibility for the influence that their environments have on post-release outcomes of its inhabitants. This framework evidences the benefit of investing in prison environments and programs that maintain and support a person’s ‘place’ in society, i.e., their connections, resources and abilities, for enabling reintegration and preventing return to prison. This would encourage changes towards more preventative health screening and care, investment into maintaining social networks outside of prison, structuring prison spaces and routines to scaffold more instrumental and independent activities such as cooking and financial management, and education regarding technological advancements. Such efforts may already be occurring sporadically across prisons or by staff who are increasingly recognizing the benefit of doing so, but a larger-scale commitment to, and enforcement of, such policies is needed.

Following this, the framework suggests a need for more active involvement of health and social services in the community to provide additional support necessary for successful reintegration of an older person during their immediate release period. The provision of basic and urgent crisis needs are a priority for this group during this time, as well as psychosocial supports to navigate a volatile time. Importantly, ‘crisis’ mode (and to a lesser extent ‘survival and adjustment’ mode) appears best supported with individualized case management. Whilst family and social supports are valuable, there is evidence that high reliance on family members for support can rather create additional stress and strain on the relationship. For those tasks specifically with working with this population, a number of social work-based practice models appear relevant, such as the Crisis Intervention Model [60] which involves key steps such as rapid assessment, establishing rapport, and exploring safe coping methods. However, in line with the general trend towards more eclectic approaches to social work [61], an integrative approach that brings together multiple methods such as that of both clinical and social work and criminal rehabilitation, would be most applicable to this unique group.

Some of the need for this case management can be offset by stronger release planning. Release planning for those whose release is imminent should at minimum cover ‘crisis mode’ needs, such that a person is equipped as well as possible to access supports and manage the volatility of this period. Programs that target the maintenance of health and key societal functions (i.e., in the ‘survival and adjustment’ mode) should also be delivered, but can occur more so during imprisonment rather than in the lead up to release. In this sense, much of a person’s sentence could be dedicated to re-equipping them for life after release.

The need for ‘better’ release planning is not a new suggestion in literature. However, changes may be difficult to enact for several reasons, such as a lack of clear quantitative data on the cost and benefits of release planning on key outcomes, or clear evidence-based frameworks upon which to scope release planning. These changes will likely involve a significant shift in the philosophy underlying the role of prisons, and decisions will ultimately reflect priorities for deprivation of liberty, security, rehabilitation or punishment. This study offers a conceptual framework that can underpin the development of such initiatives. In addition to identifying areas of need (the ‘what’), it can be used to understand the ‘how’ and ‘why’ for explaining and measuring success.

Finally, macro-level factors remain highly influential in reintegration. Whilst these are not easily tackled, long-term advocacy should still be pursued. In particular, stigma is hugely influential, with past research consistently revealing the ‘double stigma’ attached to previous incarceration status and age, that creates significant barriers for post-release support [62].

### Limitations and further work

This framework is only preliminary, and should be considered a structured qualitative analysis of the experiences of this population, that bring useful theoretical insights and practical implications. Refinements and advancements are welcome to further develop the framework, as are adaptations to fit a diverse range of people who experience incarceration. The framework in its present state is limited by the characteristics of the population in the underlying data. For this study, that was primarily people who had left prison after age 50 (45 if Aboriginal or Torres Strait Islander person) and in Australia. The framework can be improved in robustness and generalisability through additional quantitative and qualitative research to ‘test’ elements of the framework with a larger sample of older people, as well as the stakeholders and individuals who support them. Also, further consideration of other countries, and subgroups with characteristics that would entail a more unique reintegration experience, would clarify the scope of framework’s relevance. This could include younger groups or people with shorter sentence lengths, as well as a focus on women, who are a growing minority in prisons.

Furthermore, we recommend that additional research is based on an explanatory model of science (i.e., a focus on explaining phenomena, not merely describing or ordering empirical data). This would facilitate a more in depth, meaningful and rigorous analysis (and expansion) of the theoretical explanations underpinning the framework (such as “de-institutionalisation”). Further work could include clearer operationalization of the time spent within each of the stages, which would provide highly useful data for targeted intervention development.

Most importantly, refinement of the framework must be built on further input from those with lived experience and stakeholders who have supported such individuals. Whilst the current study was based on such data, these voices should be reconsulted regarding whether the resultant framework does reflect their experiences. They should play an active role in designing future iterations of the framework and all resulting interventions.

Another limitation arising from the data was that our model was focused on the earlier stages of release and reintegration. A novel contribution was made in delineating the substages that occur during this period. However, in addition to this, a desistance mindset would require more scrutiny of the long-term adjustment stage. This could occur via more consultation with people who have successfully desisted and their support networks, or those who return to prison after extended periods of adjustment.

Future research could also expand on the circumstances that lead to unsuccessful reintegration and related potential negative outcomes of failing to meet place finding needs at each stage. This would not interfere with the strengths-based nature of the framework, rather, it could provide useful indications of where and for whom current practices are failing to meet key reintegration needs, and risk of negative outcomes is highest. These should be phrased as complementary but opposite to the place finding needs of each stage. For example, relevant indicators to consider during crisis mode would include homelessness or lack of identification documents.

Additional quantitative research will be needed to assess need levels and target solutions appropriately. There is a lack of quantitative data regarding outcomes for this group in general. The current framework offers a useful guide for the types of outcomes to be considered. Longitudinal and causal models can provide valuable evidence for further development of this framework.

## Conclusions

The ‘Place-finding” framework of reintegration offers a holistic, preventative, strengths-based framework for understanding and improving reintegration in older people leaving prison. It provides a preliminary guide for both prisons and the community to respond appropriately to the rapid ageing of older people leaving prison, who are often highly marginalised and subject to health and social inequities. According to this framework, reintegration is achieved via understanding and supporting a person’s journey of ‘place-finding’, at relevant stages of their reintegration experience. The framework is aligned with sociological and criminological concepts, and offers unique theoretical contributions in suggesting a staged approach and conceptualises reintegration as a process of de-institutionalising and reconnecting individuals until they find their place in society. This framework reveals that prison environments are highly influential in post-release outcomes, and thus have a larger role to play in successful reintegration than previously acknowledged in literature, models or theories. Moreover, community-based supports and services built on social work principles that target individuals in their immediate release period are urgently needed.

## References

[1] Ginnivan NA, Butler TG, Withall AN. The rising health, social and economic costs of Australia’s ageing prisoner population. Med J Aust. 2018;209(10):422–4. doi: 10.5694/mja18.00266 30176791

[2] Bor JS. The aging of the US prison population: a public health crisis. Health Aff (Millwood). 2022;41(5):622–7. doi: 10.1377/hlthaff.2022.00280 35500173

[3] Luallen J, Cutler C. The growth of older inmate populations: how population aging explains rising age at admission. J Gerontol B Psychol Sci Soc Sci. 2017;72(5):888–900. doi: 10.1093/geronb/gbv069 26307485

[4] Yukhnenko D, Sridhar S, Fazel S. A systematic review of criminal recidivism rates worldwide: 3-year update. Wellcome Open Res. 2019;4:28. doi: 10.12688/wellcomeopenres.14970.1 31544154 PMC6743246

[5] Merkt H, Haesen S, Meyer L, Kressig RW, Elger BS, Wangmo T. Defining an age cut-off for older offenders: a systematic review of literature. Int J Prison Health. 2020;16(2):95–116. doi: 10.1108/IJPH-11-2019-0060 33634649

[6] Australian Institute of Health and Welfare. Health and ageing of Australia’s prisoners. Canberra: Australian Institute of Health and Welfare; 2019.

[7] Greene M, Ahalt C, Stijacic-Cenzer I, Metzger L, Williams B. Older adults in jail: high rates and early onset of geriatric conditions. Health Justice. 2018;6(1):3. doi: 10.1186/s40352-018-0062-9 29455436 PMC5816733

[8] Di Lorito C, Vӧllm B, Dening T. Psychiatric disorders among older prisoners: a systematic review and comparison study against older people in the community. Aging Ment Health. 2018;22(1):1–10. doi: 10.1080/13607863.2017.1286453 28282734

[9] Combalbert N, Pennequin V, Ferrand C, Armand M, Anselme M, Geffray B. Cognitive impairment, self-perceived health and quality of life of older prisoners. Crim Behav Ment Health. 2018;28(1):36–49. doi: 10.1002/cbm.2023 28276180

[10] McCausland R, Baldry E. Who does Australia lock up? The social determinants of justice. Int J Crime Justice Soc Democr. 2023. doi: 10.5204/ijcjsd.2504

[11] Baranyi G, Fazel S, Langerfeldt SD, Mundt AP. The prevalence of comorbid serious mental illnesses and substance use disorders in prison populations: a systematic review and meta-analysis. Lancet Public Health. 2022;7(6):e557–68. doi: 10.1016/S2468-2667(22)00093-7 35660217 PMC9178214

[12] Serra RM, Ribeiro LC, Ferreira JBB, Santos LL dos. Prevalence of chronic noncommunicable diseases in the prison system: a public health challenge. Ciênc saúde coletiva. 2022;27(12):4475–84. doi: 10.1590/1413-812320222712.10072022en 36383861

[13] Stürup-Toft S, O’Moore EJ, Plugge EH. Looking behind the bars: emerging health issues for people in prison. Br Med Bull. 2018;125(1):15–23. doi: 10.1093/bmb/ldx052 29394343

[14] Simpson PL, Guthrie J, Jones J, Butler T. Identifying research priorities to improve the health of incarcerated populations: results of citizens’ juries in Australian prisons. Lancet Public Health. 2021;6(10):e771–9. doi: 10.1016/S2468-2667(21)00050-5 34115972

[15] Avieli H, Band-Winterstein T. The multiple punishment of being an older adult coping with health problems in prison. Gerontologist. 2024;64(1):gnad030. doi: 10.1093/geront/gnad030 36943327

[16] Munday D, Leaman J, O’Moore É, Plugge E. The prevalence of non-communicable disease in older people in prison: a systematic review and meta-analysis. Age Ageing. 2019;48(2):204–12. doi: 10.1093/ageing/afy186 30590404

[17] Hayes AJ, Burns A, Turnbull P, Shaw JJ. Social and custodial needs of older adults in prison. Age Ageing. 2013;42(5):589–93. doi: 10.1093/ageing/aft066 23793783

[18] Field C, Archer V. Comparing health status, disability, and access to care in older and younger inmates in the New South Wales corrections system. Int J Prison Health. 2019;15(2):153–61. doi: 10.1108/IJPH-04-2018-0017 31172851

[19] Sullivan V, Forsyth K, Hassan L, O’Hara K, Senior J, Shaw J. ‘You can’t have them in here’: experiences of accessing medication among older men on entry to prison. Ageing Soc. 2015;36(06):1254–71. doi: 10.1017/s0144686x15000331

[20] Bedard R, Huxley-Reicher Z, Burke K, MacDonald R, Yang P. Aging in jail: retrospective analysis of older patients in New York City’s jail system, 2015–19. Health Aff. 2022;41:732–40. 10.1377/hlthaff.2021.01518 35500184

[21] Davoren M, Fitzpatrick M, Caddow F, Caddow M, O’Neill C, O’Neill H, et al. Older men and older women remand prisoners: mental illness, physical illness, offending patterns and needs. Int Psychogeriatr. 2015;27(5):747–55. doi: 10.1017/S1041610214002348 25428523 PMC4409101

[22] Hagos AK, Withall A, Ginnivan NA, Snoyman P, Butler T. Barriers and enablers to health and social services for older prisoners transitioning to community. Int J Prison Health. 2022;18(2):124–37. doi: 10.1108/IJPH-08-2021-0088 38899607

[23] O’Hara K, Forsyth K, Senior J, Stevenson C, Hayes A, Challis D, et al. ‘Social services will not touch us with a barge pole’: social care provision for older prisoners. J Forensic Psychiatry Psychol. 2015;26(2):275–81. doi: 10.1080/14789949.2014.1000938

[24] Poulin LIL, Colibaba A, Skinner MW, Balfour G, Byrne D, Dieleman C. Lost in transition? Community residential facility staff and stakeholder perspectives on previously incarcerated older adults’ transitions into long-term care. BMC Geriatr. 2023;23(1):180. doi: 10.1186/s12877-023-03807-3 36978019 PMC10045254

[25] Forsyth K, Daker-White G, Archer-Power L, Senior J, Edge D, Webb RT, et al. Silos and rigid processes: barriers to the successful implementation of the older prisoner health and social care assessment and plan. Med Sci Law. 2023;63(4):272–9. doi: 10.1177/00258024221141641 36448196 PMC10498653

[26] Hwang YI (Jane), Withall A, Hampton S, Snoyman P, Forsyth K, Butler T. [Preprint] Multi-sector stakeholder consensus on tackling the complex health and social needs of the growing population of people leaving prison in older age. medRxiv. 2023. doi: 10.1101/2023.04.27.23289227 PMC11027373 38639865

[27] Withall A, Mantell R, Hwang YI, Ginnivan N, Baidawi S. Background paper: issues facing older people leaving prison. 2022.

[28] Burke G, Prunhuber P, Phan T, Takshi S. Reducing barriers to reentry for older adults leaving incarceration. 2022.

[29] Australian Institute of Health and Welfare. Health and ageing of Australia’s prisoners. Canberra: Australian Capital Territory; 2019. Available from: https://www.aihw.gov.au/getmedia/1656721b-e93e-42f2-8f2c-3d6fafe647e6/aihw-phe-269.pdf.aspx?inline=true

[30] Ginnivan NA, Chomik R, Hwang YIJ, Piggott J, Butler T, Withall A. The ageing prisoner population: demographic shifts in Australia and implications for the economic and social costs of health care. Int J Prison Health. 2022;18(4):325–34. doi: 10.1108/IJPH-09-2020-0062 38899621

[31] Withall A, Mantell R, Hwang JYI, Ginnivan N, Baidawi S. Background paper: issues facing older people who are leaving prison. 2022.

[32] Hagos AK, Withall A, Ginnivan NA, Snoyman P, Butler T. Barriers and enablers to health and social services for older prisoners transitioning to community. Int J Prison Health. 2022;18(2):124–37. doi: 10.1108/IJPH-08-2021-0088 38899607

[33] Hwang YIJ, Hagos A, Withall A, Hampton S, Snoyman P, Butler T. Population ageing, incarceration and the growing digital divide: understanding the effects of digital literacy inequity experienced by older people leaving prison. PLoS One. 2024;19(4):e0297482. doi: 10.1371/journal.pone.0297482 38630834 PMC11023396

[34] Jabareen Y. Building a conceptual framework: philosophy, definitions, and procedure. Int J Qual Methods. 2009.

[35] Bronfenbrenner U. Toward an experimental ecology of human development. Am Psychol. 1977;32(7):513–31. doi: 10.1037/0003-066x.32.7.513

[36] Jimenez LB, Cross SH, Boucher NA. “He needed just about everything”: caring for aging adults postincarceration. J Appl Gerontol. 2021;40(12):1828–36. doi: 10.1177/0733464821990511 33554719

[37] Onyeali R, Howell BA, McInnes DK, Emerson A, Williams ME. The case for transitional services and programs for older adults reentering society: a narrative review of US departments of correction and recommendations. Int J Prison Health. 2023;19(1):4–19. doi: 10.1108/IJPH-08-2021-0073 36757114 PMC10123961

[38] Lares LA, Montgomery S. Psychosocial needs of released long-term incarcerated older adults. Crim Justice Rev. 2020;45(3):358–77. doi: 10.1177/0734016820913101

[39] Wyse J. Older men’s social integration after prison. Int J Offender Ther Comp Criminol. 2018;62(8):2153–73. doi: 10.1177/0306624X16683210 29770744 PMC5962031

[40] Balio CP, Norwood C, McFarlane T, Rusyniak D, Blackburn J. Health care and behavioral service use by medicaid-enrolled adults after release from incarceration. Psychiatr Serv. 2023;74(2):192–6. doi: 10.1176/appi.ps.202200035 35855622

[41] Barry LC, Steffens DC, Covinsky KE, Conwell Y, Li Y, Byers AL. Increased risk of suicide attempts and unintended death among those transitioning from prison to community in later life. Am J Geriatr Psychiatry. 2018;26(11):1165–74. doi: 10.1016/j.jagp.2018.07.004 30146371 PMC6425485

[42] Chandler MA, Van Sluytman L, Tirmazi MT, Liao M. The road home: predictors of health care utilization among older returning African American men. Rev Black Political Econ. 2020;48(1):74–92. doi: 10.1177/0034644620967002

[43] Williams BA, McGuire J, Lindsay RG, Baillargeon J, Cenzer IS, Lee SJ, et al. Coming home: health status and homelessness risk of older pre-release prisoners. J Gen Intern Med. 2010;25(10):1038–44. doi: 10.1007/s11606-010-1416-8 20532651 PMC2955468

[44] Mathlin G, Freestone M, Jones H. Factors associated with successful reintegration for male offenders: a systematic narrative review with implicit causal model. J Exp Criminol. 2022:1–40. doi: 10.1007/s11292-022-09547-5 36618557 PMC9803884

[45] Andersen TS, Scott DAI, Boehme HM, King S, Mikell T. What matters to formerly incarcerated men? Looking beyond recidivism as a measure of successful reintegration. Prison J. 2020;100(4):488–509. doi: 10.1177/0032885520939295

[46] Donnelly J. Rethinking reentry: a look at how risk-based approaches limit reentry success, and a case for why strengths-based approaches may better reduce recidivism. University of Pittsburgh; 2021.

[47] Fox KJ. Desistance frameworks. Aggress Violent Behav. 2022;63:101684. doi: 10.1016/j.avb.2021.101684

[48] Payne S. Grounded theory. Analysing qualitative data in psychology. In: Lyons E, Cole A, editors. 3rd ed. London, UK: Sage Publications; 2021. p. 196–222.

[49] Singh S, Estefan A. Selecting a grounded theory approach for nursing research. Glob Qual Nurs Res. 2018;5(2333393618799571): eCollection 2018 Jan-Dec. doi: 10.1177/2333393618799571 PMC6174650 30306099

[50] Tracy SJ. Qualitative quality: eight “big-tent” criteria for excellent qualitative research. Qual Inq. 2010;16(10):837–51. doi: 10.1177/1077800410383121

[51] Timonen V, Foley G, Conlon C. Challenges when using grounded theory. Int J Qual Methods. 2018;17(1). doi: 10.1177/1609406918758086

[52] Hwang YIJ, Hagos AK, Harris-Roxas B, Withall AL, Butler TG, Hampton S, et al. “Equipping and enabling” health literacy during a “time of change”: understanding health literacy and organisational health literacy responsiveness for people leaving prison in later life. Health Justice. 2025;13(1):21. doi: 10.1186/s40352-025-00328-6 40172730 PMC11963504

[53] Colibaba A, Skinner MW, Balfour G, Byrne D, Dieleman C. Community reintegration of previously incarcerated older adults: exploratory insights from a Canadian community residential facility program. J Aging Soc Policy. 2023;35(4):521–41. doi: 10.1080/08959420.2022.2029269 35109773

[54] Poulin LIL, Colibaba A, Skinner MW, Balfour G, Byrne D, Dieleman C. Lost in transition? Community residential facility staff and stakeholder perspectives on previously incarcerated older adults’ transitions into long-term care. BMC Geriatr. 2023;23(1):180. doi: 10.1186/s12877-023-03807-3 36978019 PMC10045254

[55] Hwang YIJ, Hampton S, Withall AL, Snoyman P, Forsyth K, Butler T. Multi-sector stakeholder consensus on tackling the complex health and social needs of the growing population of people leaving prison in older age. Health Justice. 2024;12(1):17. doi: 10.1186/s40352-024-00271-y 38639865 PMC11027373

[56] McGee TR, Farrington DP. Developmental and life-course theories of crime. In: Criminology and criminal justice. Oxford University Press; 2019. doi: 10.1093/acrefore/9780190264079.013.250

[57] Link NW, Ward JT, Stansfield R. Consequences of mental and physical health for reentry and recidivism: Toward a health‐based model of desistance. Criminology. 2019;57(3):

[58] Bonta J, Andrews D. Risk-need-responsivity model for offender assessment and rehabilitation. Rehabilitation. 2007;6:1–22.

[59] Ward T, Brown M. The good lives model and conceptual issues in offender rehabilitation. Psychol Crime Law. 2004;10(3):243–57. doi: 10.1080/10683160410001662744

[60] Roberts AR, Ottens AJ. The seven-stage crisis intervention model: A oad map to goal attainment, problem solving, and crisis resolution. Brief Treat Crisis Interv. 2005;5(4):

[61] Barth, F. D. (2014). Integrative clinical social work practice: A contemporary perspective. Springer Science + Business Media. doi: 10.1007/978-1-4939-0351-1

[62] Seaward H, Dieffenbacher S, Gaab J, Graf M, Elger B, Wangmo T. Stigma management during reintegration of older incarcerated adults with mental health issues: a qualitative analysis. Int J Law Psychiatry. 2023;89:101905. doi: 10.1016/j.ijlp.2023.101905 37329868

